# Rhabdomyolysis and Acute Kidney Injury: A Retrospective Cohort Study at a Tertiary University Hospital

**DOI:** 10.14740/jocmr6533

**Published:** 2026-04-15

**Authors:** Yannik Brien, Oliver Ritter, Daniel Patschan

**Affiliations:** a Department of Internal Medicine 1–Cardiology, Angiology, Nephrology, Pneumology, Brandenburg University Hospital, Brandenburg Medical School (Theodor Fontane), Brandenburg, Germany

**Keywords:** Rhabdomyolysis, AKI, KRT, Myoglobin, Urine pH

## Abstract

**Background:**

Rhabdomyolysis is a serious condition caused by rapid skeletal muscle breakdown, releasing substances like creatine kinase (CK) and myoglobin into the blood. If not recognized or treated, it can lead to acute kidney injury (AKI), which significantly increases health risks. This study systematically examines the epidemiology, causes, and key kidney-related outcomes of rhabdomyolysis at a tertiary university hospital.

**Methods:**

We performed a retrospective observational cohort study at the University Hospital Brandenburg, Germany, including hospitalized patients from January 1, 2023, to December 31, 2024. Inclusion required a total CK activity ≥ 500 U/L, a threshold reported in the literature to indicate increased risk for clinically relevant kidney dysfunction. Clinical endpoints were AKI as defined by the Kidney Disease: Improving Global Outcomes (KDIGO) criteria (2012), the need for kidney replacement therapy (KRT) and recovery of kidney function (ROKF).

**Results:**

Of 100 included patients (mean age 67.1 ± 19.4 years; 37% female), 60% developed AKI (KDIGO 1: 25.0%, 2: 36.7%, 3: 38.3%). AKI was significantly associated with higher age, cardiovascular/metabolic comorbidities, and elevated inflammatory and myoglobin markers. Lower urine pH in AKI patients suggests a role for aciduria in renal injury. While 49% of AKI patients showed renal recovery, 12% of the total cohort required KRT. KRT requirement was associated with younger age and higher creatinine levels, but not with peak CK or myoglobin values. Absent or incomplete renal recovery was significantly linked only to higher initial and peak creatinine levels.

**Conclusions:**

In this tertiary university care cohort, AKI was common among rhabdomyolysis patients, especially older individuals and those with cardiovascular or metabolic conditions. Inflammatory markers, myoglobin levels, and urine pH were key predictors of AKI. Despite intensive care, many required KRT and experienced incomplete renal recovery. Early diagnosis, risk stratification, and standardized criteria are needed to improve outcomes.

## Introduction

Rhabdomyolysis is a clinical syndrome characterized by acute breakdown of striated skeletal muscle with subsequent release of intracellular components such as creatine kinase (CK), myoglobin, potassium, phosphate, and uric acid into the systemic circulation [1]. Although potentially reversible, untreated rhabdomyolysis may lead to severe and life-threatening complications, most notably acute kidney injury (AKI) [2].

Despite its clinical significance, there is no universally accepted definition of rhabdomyolysis, which substantially limits comparability across studies and complicates clinical decision-making. Epidemiological data are therefore heterogeneous and likely underestimate the true incidence. Earlier estimates from the United States suggested approximately 26,000 new cases annually, with non-traumatic causes now surpassing traumatic etiologies [3]. Men are affected two to four times more frequently than women, with incidence peaks in middle-aged adults as well as in young children and elderly populations [3].

The clinical presentation is highly variable, ranging from asymptomatic elevations of CK to fulminant multiorgan failure. The classical triad of myalgia, muscle weakness, and dark urine is observed in only about 10% of cases [4, 5], whereas up to 50% of patients may be nearly asymptomatic [6, 7]. This nonspecific presentation often leads to delayed diagnosis, frequently only after complications have occurred.

AKI represents the most clinically relevant complication of rhabdomyolysis. Reported AKI rates range from 13–15% in general cohorts to as high as 65% in intensive care populations [8–10]. AKI is associated with increased mortality, prolonged hospitalization, and substantial healthcare resource utilization [11]. Electrolyte disturbances such as hyperkalemia, hypocalcemia, and hyperphosphatemia further contribute to morbidity and mortality.

The objective of this study is to systematically evaluate the epidemiology and clinical outcomes of rhabdomyolysis in a tertiary university hospital setting. The study specifically examined the following clinical outcomes: AKI, the necessity for kidney replacement therapy (KRT), and recovery of kidney function (ROKF). This project should be understood within a broader framework. At Brandenburg Medical School, the main scientific focus is on health services research. Therefore, this study was developed as an initial step toward potentially enhancing future quality of care.

## Materials and Methods

### Study design and setting

This is a retrospective observational cohort study. Data were collected from patients at the University Hospital Brandenburg (Brandenburg, Germany). The study period was from January 1, 2023, to December 31, 2024. Data collection took place from September to December 2025. Ethical approval was granted by the Ethics Committee of the Brandenburg Medical School (309052025-BO-E-RETRO). Due to the retrospective nature of the study, it was not necessary to obtain written consent from the patients. The study complies with the Declaration of Helsinki.

### Study population

Patients were identified using a code list provided by the Medical Controlling Department. The diagnosis of rhabdomyolysis was based on a pathological increase in total CK activity. The official cut-off value for CK activity was set at 500 U/L. The rationale for this chosen cut-off was based on Gaik et al [3], who postulated that clinically significant kidney dysfunction typically occurs above this value. Exclusion criteria included suspected or proven cases of myocardial ischemia, terminal illness with palliative care, outpatient care in the emergency room, chronic dialysis, and pregnancy.

### Data collection

All included patients were anonymized. Sixty-eight different parameters were collected. These included patient-related information such as age, date of birth, gender, body mass index (BMI), admission and discharge diagnosis, length of hospital stay, and possible cause of death. In addition, the following rhabdomyolysis-associated parameters were documented: total CK initial value, total CK maximum, initial myoglobin, maximum myoglobin, initial aspartate aminotransferase (AST), maximum AST, initial alanine aminotransferase (ALT), maximum ALT, etiology of rhabdomyolysis if determinable (selection between different categories such as drug-induced, traumatic, infectious, and others), therapy and resolution of rhabdomyolysis at discharge, whereby it was checked whether the CK had fallen by 50% from the maximum value. Furthermore, parameters for urine diagnostics were collected, including the presence of blood in the urine, the color of the urine, and the pH level of the urine. The following in-hospital comorbidities were documented: ventilation therapy, vasopressor therapy, sepsis and its origin, AKI during the hospital stay, AKI severity and cause, KRT used because of AKI, and ROKF. In addition, various laboratory parameters were determined with initial value, maximum value, and at discharge/death: estimated glomerular filtration rate (eGFR), sodium, serum creatinine, C-reactive protein (CRP), procalcitonin (PCT). For instrumental diagnostics, chest X-ray/chest computed tomography (CT) and transthoracic echocardiography (TTE) were performed within ± 1 day of the maximum CK value. Finally, patients were determined for the prevalence of several comorbidities: arterial hypertension, heart failure, chronic obstructive pulmonary disease, bronchial asthma, diabetes mellitus, obesity, hyperuricemia, NPL, and smoking.

### AKI, need for KRT and ROKF

AKI was defined in accordance with the Kidney Disease: Improving Global Outcomes (KDIGO) guidelines (last updated in 2012) [12]. Criteria 1 and 2 were primarily taken into account, as the third criterion (urine output) often could not be validly determined due to insufficient documentation. The endpoint of KRT was considered to have been met if at least one intermittent or continuous treatment was performed. The following criteria apply: reduced excretory kidney function accompanied by at least one of these symptoms: persistent fluid overload with uncontrollable high blood pressure or impaired oxygenation; persistent high potassium levels; worsening metabolic acidosis (venous pH below 7.15) even after giving sodium bicarbonate; or neurological symptoms not explained by anything other than elevated blood urea nitrogen. Since retention markers like blood urea nitrogen do not always match up with how severe a patient’s symptoms are, no strict values were defined that automatically triggered the use of KRT. Dialysis was carried out if a patient had no urine output for more than 24 h. Decisions were never based on CK or myoglobin concentrations. The final decision on the start of dialysis was made on an individualized basis by the responsible nephrologist. The procedures used varied between hemodialysis (HD), hemodiafiltration (HDF), slow extended daily dialysis (SLEDD), continuous veno-venous hemodiafiltration (CVVHDF). The choice of anticoagulant and the determination of blood flow rates and ultrafiltration targets were individualized based on the clinical requirements of each patient. The ROKF was defined based on serum creatinine concentration over time. In instances where the last recorded value did not differ from the lowest value by more than 10%, complete recovery was assumed. Conversely, if the difference fell between 10% and 20%, incomplete ROKF was postulated. In instances where the discrepancy exceeded 20%, it was not feasible to anticipate a ROKF. Patients who were still dependent on dialysis therapy at the time of discharge were also assigned to the “no ROKF” category.

### Statistical analysis

Categorial data were analyzed using the Chi-square test. To assess the normality of numerical data, the Shapiro–Wilk test was employed. When comparing two groups, the Mann–Whitney U test was used, while comparisons involving three or more groups were handled with the Kruskal–Wallis test. Test statistics were interpreted according to each test’s distribution; notably, the Kruskal–Wallis test follows an approximate Chi-square distribution. Analytical results included mean, median, standard deviation, U, z, both asymptotic and exact P values, as well as r values. A significance threshold of P = 0.05 was set for all analyses. For significant findings in descriptive statistics, confidence intervals were calculated. P values resulted from comparing medians and interquartile ranges (IQR). All results are presented as mean and standard deviation. All analyses were performed using Wizard for Mac OS (version 2.0.20; developer Evan Miller) and numiqo software (numiqo: Online Statistics Calculator; numiqo e.U., Graz, Austria).

## Results

### Study population

A total of 100 inpatients were included. The mean age of the entire cohort was 67.1 ± 19.4 years. The minority of patients were female (37%). The mean length of hospital stay was 17.4 ± 14.1 days. The mean BMI was 28.35 ± 7.1 kg/m^2^. Table 1 summarizes all essential clinical and laboratory characteristics of the study cohort.

**Table 1:** Baseline Characteristics of All Included Subjects

Variable	Result
Gender (female), %	37
Age (years), mean ± SD	67 ± 19.4
BMI (kg/m^2^), mean ± SD	28.3 ± 7.1
In-hospital stay (days), mean ± SD	17.4 ± 14.1
In-hospital survival, %	81
Days between admission and diagnosis of rhabdomyolysis, mean ± SD	0.6 ± 0.9
Rhabdomyolysis–etiology, %	
Ischemia	23.6
Trauma	10.9
Infection	9.1
Drug-associated (statin-associated)	7.3 (27.4% of drug-associated cases)
Autoimmune-mediated	3.6
Unknown	45.5
Rhabdomyolysis–management, %	
Volume administration	13.73
Surgery	7.84
Antibiotics	1.96
Combined	68.63
Resolution of rhabdomyolysis, %	58
Acute kidney injury (AKI), %	60
AKI stage, %	
I	25
II	36.7
III	38.3
AKI–etiology, %	
Drug-associated	33.3
Infection/sepsis	21.7
Post-surgery	8.3
Pre-renal	6.7
Cardiorenal	5
Obstruction	1.7
Combined	23.3
Kidney replacement therapy (KRT), %	12
KRT at discharge (alive or death), %	6
Recovery of kidney function (ROKF) (complete and incomplete), %	49
Laboratory findings	
Initial eGFR (mL/min), mean ± SD	53.3 ± 31.7
Minimal eGFR (mL/min), mean ± SD	42.7 ± 32.2
eGFR at discharge (mL/min), ± SD	66.4 ± 35.2
Initial sodium (mmol/L), mean ± SD	136.4 ± 6.5
Maximum sodium (mmol/L), mean ± SD	141.8 ± 6.5
Sodium at discharge (mmol/L), mean ± SD	138.8 ± 6
Initial CRP (mg/L), mean ± SD	81 ± 94.3
Maximum CRP (mg/L), mean ± SD	190.6 ± 237.7
At discharge CRP (mg/L), mean ± SD	56.7 ± 66.7
Initial PCT (ng/mL), mean ± SD)	8.4 ± 29.6
Maximum PCT (ng/mL), mean ± SD	9 ± 23
At discharge PCT (ng/mL), mean ± SD	1.3 ± 2.9
Initial CK (U/L)	2,987.2 ± 5,178.4
Maximum CK (U/L)	4,314.4 ± 6,029.1
Initial myoglobin (µg/L), mean ± SD	4,190.5 ± 7,257
Maximum myoglobin (µg/L), mean ± SD	4,609 ± 7,437.4
Urine pH, mean ± SD	5.7 ± 0.8
Morbidities (%)	
Arterial hypertension	85
Diabetes mellitus	34
Coronary artery disease	24
Heart failure	27
Chronic obstructive pulmonary disease	18
Obesity	37
Smoking	51
History of neoplasia	25

BMI: body mass index; eGFR: estimated glomerular filtration rate; CRP: C-reactive protein; PCT: procalcitonin; CK: creatine kinase; SD: standard deviation.

### Rhabdomyolysis: epidemiology, etiology and resolution

The average time between hospital admission and diagnosis of rhabdomyolysis was 0.6 ± 0.9 days. The most common cause was identified as ischemia (23.6%), followed by trauma (10.9%), infections (9.1%), drug effects (7.3%), and autoimmune causes (3.6%). In 45.5% of cases, the cause remained unknown. The therapeutic approach included volume replacement alone in 13.73% of cases, surgical measures in 7.84%, and the use of antibiotics in 1.96%. Combined therapeutic approaches were used most frequently (68.63%). Resolution of rhabdomyolysis was observed in 55.8% of cases (Table 1).

### AKI

**Figure 1:**
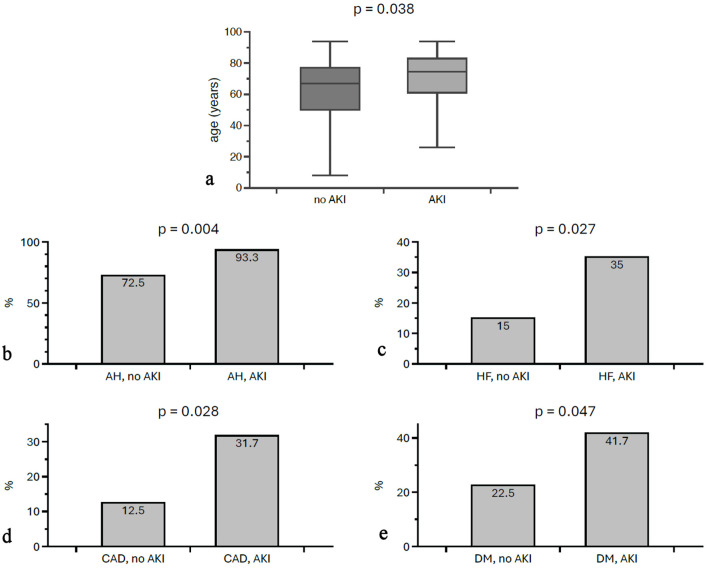
Differences in age and morbidities between individuals with and without AKI. AKI patients were older (a) and more likely to suffer from morbidities such as AH (b), HF (c), CAD (d), and DM (e). AKI: acute kidney injury; AH: arterial hypertension; HF: heart failure; CAD: coronary artery disease; DM: diabetes mellitus.

During the course of treatment, 60% (n = 60) of patients developed AKI according to KDIGO 2012 [12]. Of the patients affected, 25.0% were in stage 1 according to KDIGO, 36.7% in stage 2, and 38.3% in stage 3. With regard to etiology, a drug-related cause could be identified in 33.3% of cases. Other causes included infections (21.7%), postoperative causes (8.3%), prerenal factors (6.7%), cardiorenal syndrome (5.0%), and obstructive causes (1.7%). In 23.3% of cases, no cause could be identified. Recovery of renal function, both complete and partial, was demonstrated in 49.0% of cases (Table 1). Affected individuals were older on average than non-AKI patients (71.08 ± 15.69 vs. 61.10 ± 22.88 years, P = 0.039). In addition, there were significant differences in the prevalence of comorbidities between patients with and without AKI. Patients with AKI were significantly more likely to have arterial hypertension (93.3% vs. 72.5%; P = 0.004), coronary heart disease (31.7% vs. 12.5%; P = 0.028), and heart failure (35.0% vs. 15.0%; P = 0.027). Diabetes mellitus was also more common in this group (41.7% vs. 22.5%; P = 0.047) (Fig. 1). In supplementary multiple linear regression analyses, AKI was defined as a dependent variable, gender, the maximum CK concentration and either age, arterial hypertension, diabetes mellitus, coronary artery disease or heart failure were defined as covariables. In all analyses, the covariables remained significantly associated with AKI, except for diabetes mellitus: age (P = 0.03); arterial hypertension (P = 0.01); coronary artery disease (P = 0.01); and heart failure (P = 0.03).

**Figure 2:**
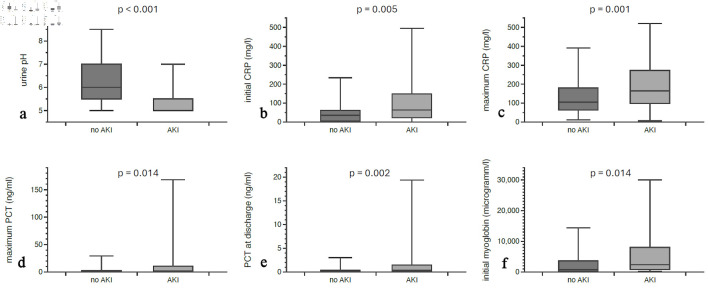
Laboratory differences between AKI patients and patients without acute kidney dysfunction. Urine pH was lower in AKI patients (a), while initial CRP (b), maximum CRP values (c), maximum PCT (d), PCT at discharge (e), and initial myoglobin (f) were significantly higher. AKI: acute kidney injury; PCT: procalcitonin; CRP: C-reactive protein.

A marked distinction was observed in urine diagnostics; specifically, patients with AKI exhibited a significantly lower urine pH (5.36 ± 0.47) compared to non-AKI individuals (6.21 ± 0.99, P < 0.001), as illustrated in Figure 2. The initial CRP value was higher in AKI (102.82 ± 109.06 mg/L vs. 47.46 ± 50.28 mg/L; P = 0.005), as was the maximum CRP (194.23 ± 132.87 mg/L vs. 135.4 ± 93.87 mg/L; P = 0.001) (Fig. 2). Significant differences were also documented for PCT: the maximum PCT was significantly elevated in patients with AKI (12.79 ± 28.16 ng/mL vs. 2.76 ± 6.05 ng/mL; P = 0.014), as was the PCT at discharge (1.85 ± 3.52 ng/mL vs. 0.42 ± 0.74 ng/mL; P = 0.002). Also, patients with AKI had significantly higher initial myoglobin levels (5,824.93 ± 9,093.1293 µg/L vs. 2,200.8 ± 3,267.68 µg/L; P = 0.041) (Fig. 2). The initial serum creatinine level in AKI patients was 217.83 ± 224.09 µmol/L compared to 86.88 ± 33.01 µmol/L in patients without AKI (P = 0.001). The difference was even more pronounced for maximum creatinine (306.27 ± 249.08 µmol/L vs. 93.27 ± 37.44 µmol/L; P = 0.001) and creatinine at discharge (162.75 ± 143.31 µmol/L vs. 70.97 ± 37.78 µmol/L; P = 0.001). The variables length of hospital stay, BMI, CRP at discharge, initial PCT levels, initial and maximum sodium levels and sodium levels at discharge, initial and maximum CK, and maximum myoglobin did not differ significantly between AKI and non-AKI groups.

### KRT

Twelve patients (12%) required KRT during the hospital stay. These patients were significantly younger (61.42 ± 9.76 years for KRT vs. 67.86 ± 20.30 years for non-KRT; P = 0.045). Patients requiring KRT showed higher initial (417.88 ± 437.29 µmol/L vs. 131.03 ± 107 µmol/L; P = 0.014), maximum (623.25 ± 392.82 µmol/L vs. 166.23 ± 101.77 µmol/L; P = 0.001) and discharge serum creatinine values (293.30 ± 242.90 µmol/L vs. 103.23 ± 71.04 µmol/L; P = 0.001). In addition, PCT at discharge was significantly elevated in patients requiring KRT (2.56 ± 2.64 ng/mL vs. 1.13 ± 2.92 ng/mL; P = 0.001). The variables length of hospital stay, BMI, initial and maximum sodium, initial and maximum CRP, CRP at discharge, initial and maximum PCT, initial and maximum CK, and initial and maximum myoglobin did not differ significantly between KRT and non-KRT groups.

### ROKF

Initial serum creatinine differed significantly among the groups “no RKF” (n = 50; median 141.5 mg/dL), “incomplete ROKF” (n = 18; median 81.75 mg/dL) and “complete ROKF” (median 112.5 mg/dL) (P < 0.001). Maximum creatinine also differed significantly among the “no,” “incomplete,” and “complete” ROKF groups (median 207.5 mg/dL vs. 90.6 mg/dL vs. 138.5 mg/dL; P < 0.001). For all other parameters examined, there were no statistically significant differences among the “no,” “incomplete,” and “complete” ROKF groups.

## Discussion

A notably high rate of AKI was observed, affecting about 60% of participants who experienced a sudden decline in kidney function. Those with AKI were older compared to those without it. The AKI group also showed lower urinary pH levels. Additionally, both initial and peak CRP readings, as well as maximum PCT values, were higher in these patients, as was initial serum myoglobin. Interestingly, individuals needing KRT were younger. Ultimately, there were no identifiable predictive factors for ROKF.

In Central European hospitals, AKI occurs in about 10–15% of patients [13]. A 60% AKI incidence, as seen in our cohort, is usually reported among intensive care patients [14]. The advanced age of those affected is not surprising in itself, as advanced age is generally considered a risk factor for acute (and chronic) kidney dysfunction [15]. The lower urine pH fits in with the concept of myoglobin-associated kidney damage. The literature still discusses the possibility of alkalization to prevent kidney damage in rhabdomyolysis patients. This is based on the assumption that raising the urine pH increases the solubility of myoglobin in the tubules. However, this concept is now viewed rather critically [16]. Elevated humoral inflammation parameters (CRP) upon hospital admission and higher maximum CRP and PCT values indicate a higher degree of cumulative morbidity in patients with AKI. These findings, combined with the notably elevated incidence of AKI, indicate that individuals experiencing rhabdomyolysis accompanied by AKI constitute a particularly high-risk population, with outcomes potentially comparable to those observed in intensive care settings. This inference is further supported by a higher prevalence of comorbidities. However, numerous parameters did not differ between AKI patients and those without AKI, including length of hospital stay, BMI, CRP at discharge, initial PCT levels, initial and maximum sodium levels and sodium levels at discharge, initial and maximum CK, and maximum myoglobin. The findings regarding CK are particularly surprising, since CK is the most sensitive marker for the occurrence of rhabdomyolysis [17]. Nevertheless, our results support the assumption that CK primarily reflects the extent of muscle damage but has only limited prognostic value with regard to kidney-related complications. Initial myoglobin levels differed between patients with and without AKI, whereas peak myoglobin values were not significantly associated. This may be due to myoglobin’s short half-life (2–3 h) [18], making it less useful than CK for detecting skeletal muscle diseases. The timing of laboratory diagnostics is therefore crucial for accurately interpreting myoglobin levels. Consequently, the assumption that an increased peak myoglobin concentration corresponds to a heightened risk of requiring dialysis should be reconsidered. Additionally, no significant correlation was observed between CK or myoglobin levels and ROKF. These results reinforce the premise that prognosis is determined more by the duration and severity of kidney injury than by the initial extent of muscle damage.

Patients who required KRT due to AKI were younger than those without KRT. At first glance, this trend seems implausible, as the extent of cumulative morbidity increases with age. However, the age difference in our cohort was only a few years (61.42 vs. 67.86 years), and only 12% of all patients required KRT. It should at least be considered that this paradoxical finding could be a reflection of the small group size. With the exception of serum creatinine measured at different times, there were in fact no differences between dialysis-dependent and non-dialysis-dependent patients in various variables (except for PCT at discharge). It should be emphasized once again that neither CK nor myoglobin differed in any way between the two groups. This observation could be relevant insofar as both parameters appear to be of little relevance when deciding whether to start dialysis or not.

It may be considered disappointing that predictors of ROKF have yet to be identified. The issue of whether, how quickly, and to what extent organ function recovers following AKI is important not only for rhabdomyolysis-associated AKI but for anyone experiencing an acute decline in kidney excretory function. There is still debate regarding the precise criteria that define ROKF, though several reviews have discussed this topic [19–21]. It is generally accepted, however, that ongoing dysfunction beyond 7 days is classified as AKD [22]. Individuals affected face different risks of developing chronic kidney disease after 4 months [23], along with related cardiovascular complications and mortality. While differences in serum creatinine levels were observed across various ROKF categories, it is clear that creatinine itself is not a reliable prognostic marker. Nonetheless, our findings indicate that the rhabdomyolysis indicators, CK and myoglobin, are insufficient for determining the probability of ROKF. Similarly, the humoral inflammation markers do not provide conclusive evidence in this context.

Finally, it would be necessary to ask whether the data collected are representative or could allow conclusions to be drawn about the etiology, course and risk profile of rhabdomyolysis in other areas of Germany or Europe. The group is largely representative of around 200,000 residents living within the catchment area. While there are certain epidemiological differences among the federal states of Germany—such as variations in age distribution, healthcare availability, and possibly quality of care—the population described still offers a reasonable reflection of both the German and, roughly, Central European populations. We regard the data as generally representative, though we remain aware of the constraints inherent to retrospective surveys.

### Conclusions

In summary, the following conclusions are evident: 1) The incidence of rhabdomyolysis-associated AKI aligns with the incidence of AKI observed in intensive care units; 2) Affected patients tend to be older; however, among those requiring dialysis, the mean age is comparatively lower; 3) The presence of AKI is associated with more pronounced, though potentially transient, humoral inflammatory responses; 4) Accurate prediction of renal outcome remains challenging in cases of rhabdomyolysis accompanied by AKI; 5) While CK and myoglobin hold limited prognostic value, these parameters may still serve a diagnostic function.

### Limitations

A fundamental limitation in the investigation of rare diseases is, of course, the small number of cases. Although 100 patients with the diagnosis could be identified, this sample size is small compared with that of large, multicenter studies, such as those in cardiovascular medicine. The retrospective design is a limitation, the reasons for which need not be explained in detail. Another limitation is the lack of follow-up data on those who were discharged from the hospital. In addition, it was not possible to secure sufficient information on the urine production of those affected, and in many cases, there was no corresponding documentation. Another limitation, which is unavoidable, is the lack of histological information on the patients. Of course, it would be extremely desirable to know to what extent intratubularly precipitated myoglobin may have caused or at least contributed to kidney damage.

## Data Availability

The data supporting the findings of this study are available from the corresponding author upon reasonable request.
